# Centuries of suffering from a preventable cause: where is that human intelligence?

**DOI:** 10.1186/1617-9625-1-1-1

**Published:** 2002-01-15

## 

The history of tobacco use dates back to 1492, when *Rodrigo de Jerez *and *Luis de Torres*, in Cuba searching for the Khan of Cathay (China), were credited with the first observing smoking. Although *Jean Nicot de Villemain*, France's ambassador to Portugal, wrote as early as in 1560 about the medicinal properties of tobacco, the pharmaceutical use of this plant is still negligible in 2002 compared to an incredibly high prevalence of tobacco misuse. The human concern to prevent the noxious effects of this plant as a drug of abuse also began early in 1603, when for the first time, British physicians demonstrated to be upset about the tobacco use without prescription. What the humans pursue now as a 'ban on smoking' is not new either; *Pope Urban VIII *was the first to ban smoking in holy places in 1642. What have we learned from all these messages from the past?

A product of the nature, a self-growing plant was discovered several centuries ago, curiosity of a few ancients led to a bad habit of dependency, some opportunists seeking profit began to cultivate the plant in great amounts, and so began an ancient addiction epidemic that soon became pandemic. The ancient rulers of those era being *unaware *of the health hazards of tobacco use found smoking a luxurious habit and thus offered their guests with tobacco leaves. A few centuries later, the modernized rulers of the millennium era in 'politician suits', although *aware *of the hazards, still found it luxurious to offer their guests with the first class *Havana *cigars and to take puffs of these themselves where cameras show up, and even believe (or are advised to believe) that a mass dependency on tobacco products is well beneficial to the economy!! The outcome: centuries of suicide and legal homicide by a deadly bad habit for the profit of some bad boys.

A few years ago, WHO has estimated that every 10 seconds, another person dies as a result of tobacco use and that the death toll increases rapidly [WHO global status report 1997]. The same agency has also predicted that due to an increasing pattern of smoking prevalence, this death toll will rise to about 10 million per year by the 2020s. These figures represent a close-to-true and unbelievably high mortality rate of humans following years of suffering from varieties of tobacco hazards. Of course, not everyone suffering from a tobacco-caused disease dies or is going to die soon, but knowing the fact that no smoker (or tobacco chewer/snuffer) is going to bypass the hazards of highly poisonous tobacco extracts or smoke constituents without being affected, the worldwide morbidity rate should at least be a sum of those data that represent "tobacco consumption prevalence" among the annual economic reports of all governments and not the data that appear on "nations' health reports".

By definition, *disease *is a condition of any deviation from the normal structure or function of the body and is manifested by a characteristic set of symptoms and signs, whose etiology, pathology, and prognosis may be known or unknown. Although there are many such conditions that have been suspected but not yet fully linked to tobacco use, numerous other illnesses have long been identified as to be either caused or worsened by tobacco use. However, how many of such diseases are known to us and for how many of these are the exact underlying mechanisms of pathogenesis identified? Of several thousands of poisonous substances that constitute cigarette smoke or tobacco extracts, only a few hundreds are known for their pathobiological effects in *in vitro *and/or *in vivo *systems, much less within the human body. Regardless of all international scientific efforts and the heavy annual budgets spent, the medical science is still slow-moving towards identification of all these health damages. This is because, there are too many poisons released by a tobacco product in a human body, which in turn cause too many damages themselves and/or through their metabolites, and there are too many interactions by the diet and other xenobiotics that make a study process complicated. However, the question arises as to why should humans use their intelligence and billions of dollars each year in order to investigate on mechanisms of actions and treatments of deadly diseases that are simply preventable?

There is almost no human organ or tissue that is not biochemically and/or functionally affected after using a tobacco product, in particular after smoking. When an adult suffers from a single or multi-organ damage(s) following smoking, this negatively affects the family's psychological and economical conditions, such ill families affect the local society's performance, which in turn negatively affect the overall social health and productivity of a nation. Simultaneously, the government benefits from a tobacco sale taxation and the bad boys widespread tobacco addiction and enjoy their profits. Consequently, such a nation deteriorates after a while as the annual expenses for the nation's health care dramatically exceed the income from revenues and the social productivity touches the bottom of its limits. The addicted jobless population caches hold of more cigarettes financed through social security aids, the government goes for lending money from tobacco producers or another addicted country, and the bad boys enjoy again their ever increasing profits. What is that all about for the sake of God? Is it too complicated to be understood by finance and health ministers? Should the politicians be tested for intelligence prior to candidacy for an office?

With the release of this issue, a new professional journal is introduced to the world's medical society. But what is unique about it? The answer to this is perhaps difficult to be comprehended by those who still neglect the above situations and the progressive, unreasonable mass mortality of humans. The answer: currently there is no high-quality journal that publishes studies in all dynamic areas of clinical, experimental, biological, psycho-social and educational research to build *a central archive *of all related '*non-industrial studies*' on tobacco health hazards. By publishing Tobacco Induced Diseases, the PTID Society will lead and create synergies in thinking about all scientific aspects of tobacco harms across adverse readership, and maximize the impact of the science on the prevention of such diseases. Supports are needed from true and independent scientific societies and the science dedicated researchers.

Ed Nelson

Essen University School of Medicine

## Competing interests

The authors declare that they have no competing interests.

